# Bioaggregate of photo-fermentative bacteria for enhancing continuous hydrogen production in a sequencing batch photobioreactor

**DOI:** 10.1038/srep16174

**Published:** 2015-11-05

**Authors:** Guo-Jun Xie, Bing-Feng Liu, Rui-Qing Wang, Jie Ding, Hong-Yu Ren, Xu Zhou, Nan-Qi Ren

**Affiliations:** 1 State Key Laboratory of Urban Water Resource and Environment, Harbin Institute of Technology, Harbin 150090, China; 2 Advanced Water Management Centre, The University of Queensland, QLD 4072, Australia

## Abstract

Hydrogen recovery through solar-driven biomass conversion by photo-fermentative bacteria (PFB) has been regarded as a promising way for sustainable energy production. However, a considerable fraction of organic substrate was consumed for the growth of PFB as biocatalysts, furthermore, these PFB were continuously washed out from the photobioreactor in continuous operation because of their poor flocculation. In this work, PFB bioaggregate induced by L-cysteine was applied in a sequencing batch photobioreactor to enhance continuous hydrogen production and reduce biomass washout. The effects of the hydraulic retention time (HRT), influent concentration and light intensity on hydrogen production of the photobioreactor were investigated. The maximum hydrogen yield (3.35 mol H_2_/mol acetate) and production rate (1044 ml/l/d) were obtained at the HRT of 96 h, influent concentration of 3.84 g COD/l, and light intensity of 200 W/m^2^. With excellent settling ability, biomass accumulated in the photobioreactor and reached 2.15 g/l under the optimum conditions. Structural analysis of bioaggregate showed that bacterial cells were covered and tightly linked together by extracellular polymeric substances, and formed a stable structure. Therefore, PFB bioaggregate induced by L-cysteine is an efficient strategy to improve biomass retention capacity of the photobioreactor and enhance hydrogen recovery efficiency from organic wastes.

Increasing concerns about fossil fuels depletion and environmental pollutions caused by continuously growing energy demands have promoted worldwide production and use of renewable energy^1^,^2^. Hydrogen is an attractive potential alternative energy source for reducing global dependence on fossil fuels and mitigating the climate footprint of economies^3^,^4^,^5^. Among the various processes, biological hydrogen production stands out as an appealing choice, as hydrogen can be produced from renewable sources by certain groups of microorganisms at ambient temperatures and normal pressures^6^,^7^,^8^. However, the major challenge for the commercialization of biological hydrogen production is low product yields, since high hydrogen yields are critical to achieve their production economics^9^,^10^,^11^,^12^. Photo-fermentative hydrogen production has the potential to increase the feedstock conversion efficiency, as it incorporates solar energy into renewable energy production from waste biomass^13^,^14^. During the photo-fermentation, photo-fermentative bacteria (PFB) capture energy from sunlight to oxidize organic compounds and generate electron potential needed to drive hydrogen production, and then nitrogenase acts as an ATP-powered hydrogenase to produce hydrogen exclusively without inhibitory feedback^15^. Through solar-driven biomass conversion, PFB could potentially divert 100% of electrons from organic substrates to hydrogen production. Therefore, photo-fermentative hydrogen production has been suggested as one of the most promising hydrogen production processes.

To achieve a rapid and stable hydrogen production, PFB are expected to be retained in the photobioreactor to produce hydrogen continuously. However, continuous hydrogen production by PFB always suffers from a serious biomass washout from photobioreactor, due to their poor flocculation. Extracellular polymeric substances (EPS) covered on the cell surface have been demonstrated to play a decisive role in the process of bioaggregate formation^16^. In order to understand the flocculation behavior of PFB, EPS production by *Rhodopseudomonas acidophila* under various conditions, relationship among EPS contents, components, and the bacterial flocculation were investigated^17^,^18^. The results showed that EPS and their contents and composition have a significant effect on the bacterial surface characteristics and flocculability. According to the DLVO theory, contribution of van der Waals interaction energy to total interaction energy could be neglected because effective Hamaker constant between *R. acidophila* and water was only 2.27 × 10^−23^ J^19^. As a result, the bacterial particles could not overcome the total energy barrier to flocculate effectively. In addition, cell particles of *R. acidophila* repelled each other due to positive total interfacial free energy between the bacterial cells and water, which resulted in a great stability of the cell suspensions. Consequently, PFB were washed out with effluent from reactor continuously in the continuous operation^20^, which contributed to effluent turbidity and led to increases in pollutants. Moreover, organic substrate was continuously utilized for cell growth to supplement biomass washout and maintain the biomass concentration in the steady state operation^20^,^21^,^22^,^23^. As a result, most of substrate (37–50%) was used for biomass synthesis^22^,^24^, while substrate used for hydrogen production only accounted for little proportion (16–45%)^23^,^25^,^26^.

In our previous study, L-cysteine was demonstrated to induce remarkable aggregation of *Rhodopseudomonas faecalis* RLD-53 and simultaneously improve hydrogen production in the batch culture^27^. Through formation of disulphide bonds which are the medium for extracellular secretion of proteins, L-cysteine not only promoted production of EPS, in particular the secretion of protein, but also stabilized the final conformation of protein in EPS^27^. With the increase of EPS covering on cell surface, cell surface properties, especially surface charged groups, have also been changed. Consequently, absolute zeta potential reached a minimum value at 1.0 g/l of L-cysteine, which indicated significant decrease of electrostatic repulsion interaction energy based on the DLVO theory. Total interaction energy barrier decreased from 389.77 *k*_*B*_*T* (*k*_*B*_, Boltzmann constant; *T*, absolute temperature) at 0.0 g/l of L-cysteine to 127.21 *k*_*B*_*T* at 1.0 g/l. Therefore, *R. faecalis* overcame the total energy barrier and flocculated effectively. After a short settlement, the biomass washout will be significantly reduced and the effluent quality will be greatly improved.

The objective of this work is to evaluate the operational stability of the bioflocculation induced by L-cysteine for the continuous hydrogen production in a sequencing batch photobioreactor. Firstly, three key operational parameters, hydraulic retention time (HRT), influent concentration and light intensity, were optimized for investigating the hydrogen production performance and biomass retention capacity in bioreactor. Then, the structure and components distribution of bacterial floc were also investigated by scanning electron microscope (SEM) and confocal laser scanning microscope (CLSM).

## Results

### Effect of HRT on the performance of PFB bioaggregate

**Figure 1:**
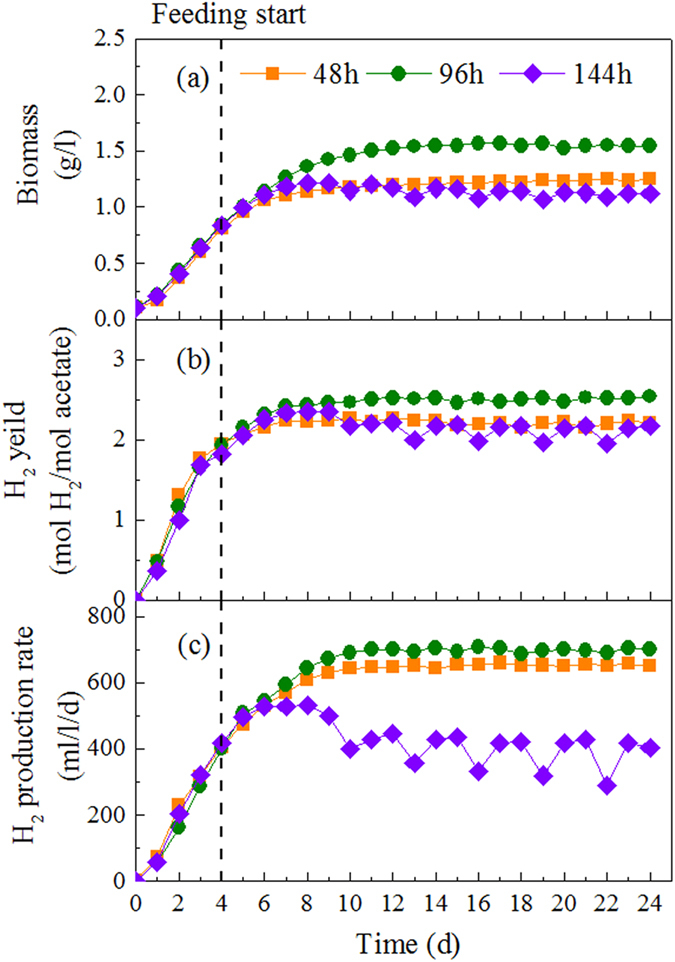
Continuous hydrogen production by bioaggregate of *R. faecalis* RLD-53 under different HRTs: (**a**), biomass concentration; (**b**), hydrogen yield; (**c**) hydrogen production rate.

**Figure 2:**
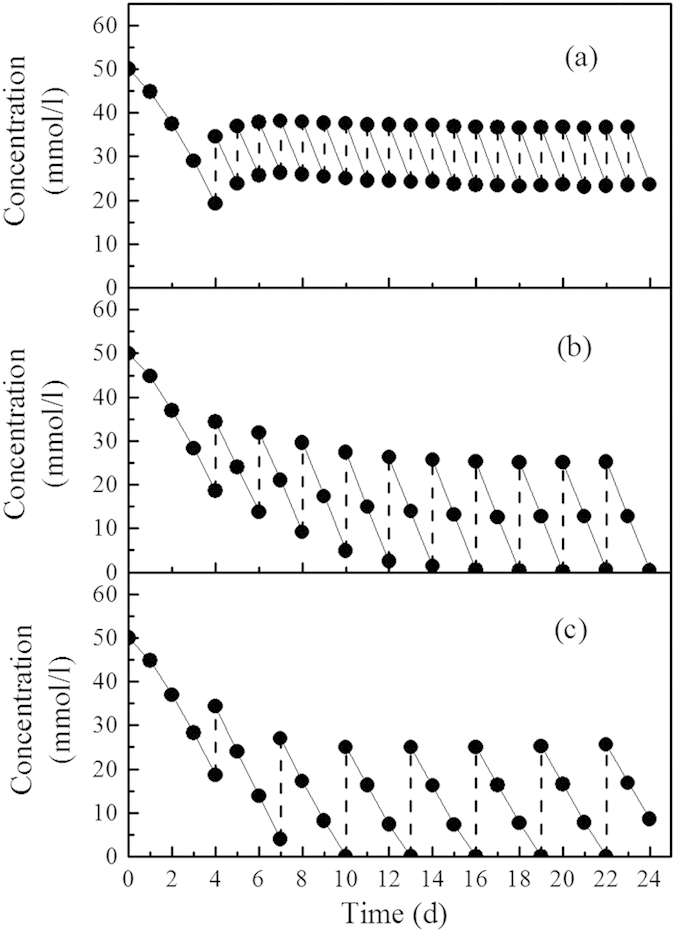
Substrate concentration in photobioreactor under different HRTs: (**a**), 48 h; (**b**), 96 h; (**c**), 144 h.

The hydrogen production performance and biomass retention capacity of the photobioreactor were influenced well by the variations of the HRT (48, 96 and 144 h) (Fig. 1). Hydrogen production rate at HRT of 144 h was fluctuating with a mean value of 395 ml/l/d, which may be due to that substrate was exhausted at the end of each cycle period (Fig. 2c). Under such conditions, poor hydrogen productivity was expected since the PFB were receiving inadequate reducing equivalents and thus producing lower amount of hydrogen per reactor volume^28^. As a result, a hydrogen yield was only 2.17 mol H_2_/mol acetate at HRT of 144 h. With the decrease of HRT to 96 h, hydrogen production rate increased up to 702 ml/l/d with the maximum hydrogen yield of 2.54 mol H_2_/mol acetate. The results indicated that shortening HRT could enhance the hydrogen production performance, due to the availability of more substrate to the PFB. Biomass concentration also achieved its maximum of 1.55 g/l at the HRT of 96 h, which consequently supported the hydrogen production. However, with the further decreasing HRT to 48 h, the biomass concentration was decreased from 1.55 g/l to 1.12 g/l, and it was obvious that shorter HRT washed out more biomass. With the decrease of hydrogen producers in the photobioreactor, hydrogen production rate dropped to 651 ml/l/d at HRT of 48 h. In addition, acetate concentration in the effluent was about 23.52 mmol/l with substrate removal efficiency only 52.96% (Fig. 2a), which indicated that a significant proportion of substrates were not converted into hydrogen but were washed away with the effluent at shorter HRT.

### Effect of influent concentration on performance of PFB bioaggregate

**Figure 3:**
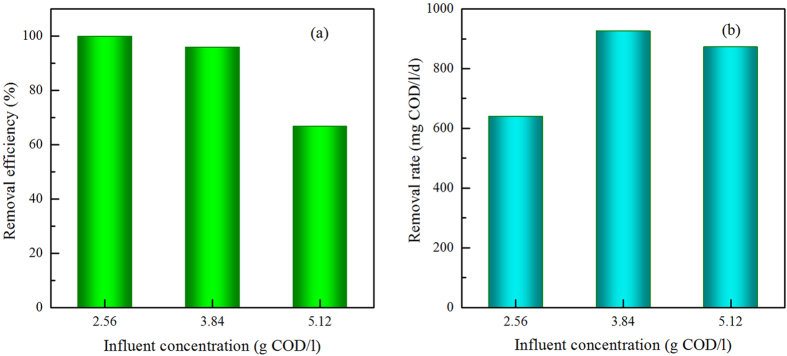
The effect of influent concentration on COD removal efficiency (a) and COD removal rate (b) at HRT of 96 h.

The effect of influent concentration on COD removal efficiency and COD removal rate were shown in Fig. 3. At influent concentration in the range of 2.56–3.84 g COD/l, acetate was converted by more than 96% (Fig. 3a). However, acetate conversion decreased to 85% at the highest influent concentration (5.12 g COD/l), suggesting that the photobioreactor was operated under an overloading condition. The COD removal rate increased with influent concentration from 2.56 to 3.84 g COD/l, and reached peak of 926 mg COD/l/d, but then slightly decreased as the influent concentration further increased to 5.12 g COD/l (Fig. 3b).

**Figure 4:**
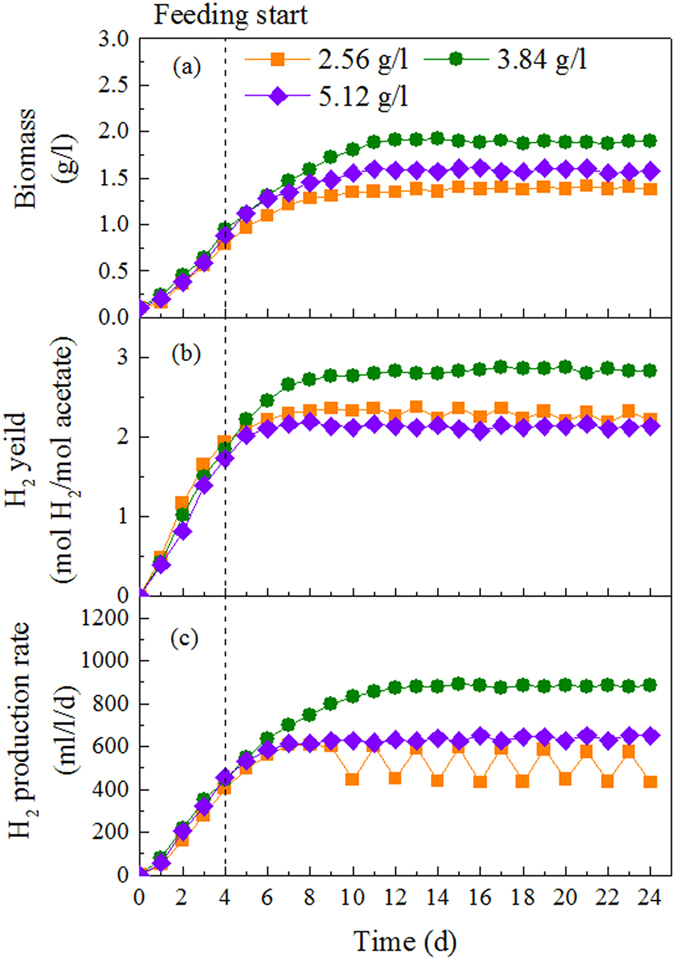
Continuous hydrogen production by bioaggregate of *R. faecalis* RLD-53 under different influent concentrations at HRT of 96 h: (a), biomass concentration; (b), hydrogen yield; (c) hydrogen production rate.

For influent concentrations between 2.56 to 3.84 g COD/l, the hydrogen production rate increased with increasing influent concentration (Fig. 4). Due to insufficient substrate at the end of each cycle period at influent concentration of 2.56 g COD/l, hydrogen production rate was unstable with an average of 504 ml/l/d. As a result, hydrogen yield was 2.19 mol H_2_/mol acetate. For an influent concentration of 3.84 g COD/l, the maximum hydrogen production rate of 886 ml/l/d was achieved with hydrogen yield of 2.82 mol H_2_/mol acetate. However, due to overloading caused by high influent concentration of 5.12 g COD/l, hydrogen production rate and yield sharply declined to 649 ml/l/d and 2.13 mol H_2_/mol acetate. The biomass concentration increased with influent concentration and reached maximum of 1.89 g/l at 3.84 g COD/l, but then decreased to 1.57 g/l as the influent concentration increased to 5.12 g COD/l.

### Effect of light intensity on performance of PFB bioaggregate

**Figure 5:**
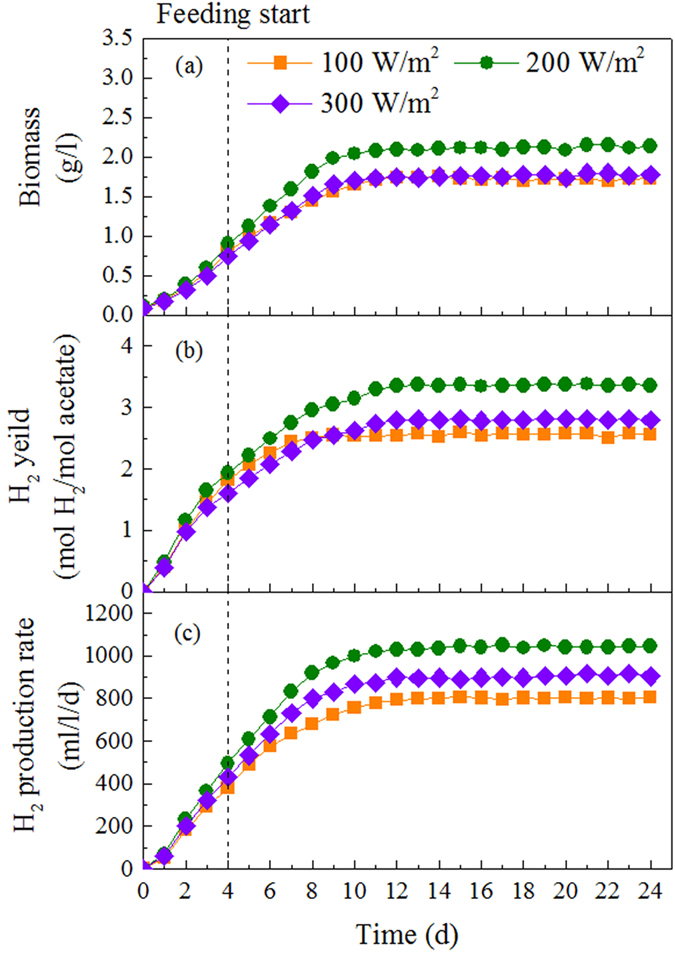
Continuous hydrogen production by bioaggregate of *R. faecalis* RLD-53 under different light intensities: (a), biomass concentration; (b), hydrogen yield; (c) hydrogen production rate.

An optimal illumination condition in the photobioreactor is essential for higher yield of hydrogen production by PFB. In this study, hydrogen production rate and hydrogen yield increased with increasing light intensity from 100 to 200 W/m^2^ (Fig. 5). At light intensity of 100 W/m^2^, the hydrogen production rate and hydrogen yield was only 806 ml/l/d and 2.56 mol H_2_/mol acetate. This was attributed to the fact that the driven force of light was weak in limited incident light intensity, which induced little excited electrons for the substrate degradation. As a result, there was high concentration of substrate in the effluent. When sufficient light energy provided (200 W/m^2^), the photosynthetic apparatus of PFB could absorb more photon to excite the biological activity and promote the substrate degradation for hydrogen production (Fig. 5). Consequently, the maximum hydrogen production rate of 1044 ml/l/d and hydrogen yield of 3.35 mol H_2_/mol acetate were observed at 200 W/m^2^, respectively. However, a further increase in light intensity from 200 to 300 W/m^2^ resulted in a decrease in hydrogen production.

## Discussion

Hydraulic retention time (HRT) is one of the most important parameters for photo-fermentative hydrogen production, as it determines the carbon source availability to photo hydrogen producers and also the washout of these producers from the photobioreactor. Long HRTs from 25 h^29^ to 120 h^30^ were usually required for photo-fermentation, because of slow conversion of organic substrates to H_2_ and CO_2_ and slow growth rate of PFB. In this study, better performance was achieved at longer HRT (144 h) in terms of organic substrate removal (Fig. 2), while the hydrogen production was limited due to insufficient carbon source supply. The unsettleable cells were removed as HRT decreased to 96 h, which favored the growth of aggregating biomass within the photobioreactor. Further shortening of the HRT (48 h) resulted in excessive hydrogen producer washout, and consequently production rate and yield of hydrogen were poor. Moreover, the reducing HRT also increase the availability of organic substrate to PFB, while low biomass concentration in reactor led to the decrease of substrate consumption. As a result, a significant amount of organic substrate was wash out at HRT of 48 h, giving the substrate removal efficiency of 52.96%.

It is essential to determine the proper range of influent concentration that the photobioreactor can handle effectively^31^, and influent concentration has also been considered as the most important parameter to control the formation of anaerobic bioaggregate^32^. When influent concentration reached 5.12 g COD/l, large numbers of planktonic cells presented in the photobioreactor, which indicated that higher influent concentration exceeded the limitation that flocs could withstand and stimulated the growth of planktonic cells. With a considerable amount of stably suspended cells discharged from the reactors, the biomass concentration decreased from 1.89 g/l at 3.48 g COD/l to 1.57 g/l at 5.12 g COD/l. It has been shown that an increased substrate concentration raises the biomass growth rate, and high growth rate of anaerobic microorganisms would reduce the strength of the three-dimensional structure of bioaggregate^32^. As a result, the bioaggregate would easily lose their structural integrity, and disintegration would occur. The disintegration of bioaggregate caused by excessive influent concentration was also observed in aerobic granular reactor^33^. When influent concentration increased to 3000 mg COD/l, the aerobic granular sludge gradually disintegrated, resulted in a large amount of flocculent sludge discharged from the reactors and eventually led to collapse of the system.

Light intensity is another important parameter that regulates the photo-fermentative hydrogen process. High illumination intensity offers more ATP and reductive power to the photosynthetic system, which are favorable for hydrogen production. However, when the photosynthetic system of PFB exposed to excessive photons under high light intensity of 300 W/m^2^, photons cannot all be used for reaction energy but are dissipated as heat energy which may damage the photosynthetic apparatus^34^. As a result, hydrogen production was decreased at high light intensity of 300 W/m^2^. In this study optimum hydrogen production was obtained under light intensity of 200 W/m^2^, which is relatively higher than solid-carrier assisted photo-fermentation with internal optical-fiber illumination^35^. The sunlight intensity during the day was very high^36^ even with the maximum of 1000 W/m^2^, which is much higher than the optimum light intensity that photo-fermentative bioaggregate required. In order to avoid light saturation effect caused by excessive sunlight, spatial dispersion of the high illumination has been developed using light shade bands covered on the photobioreactor^37^.

**Figure 6:**
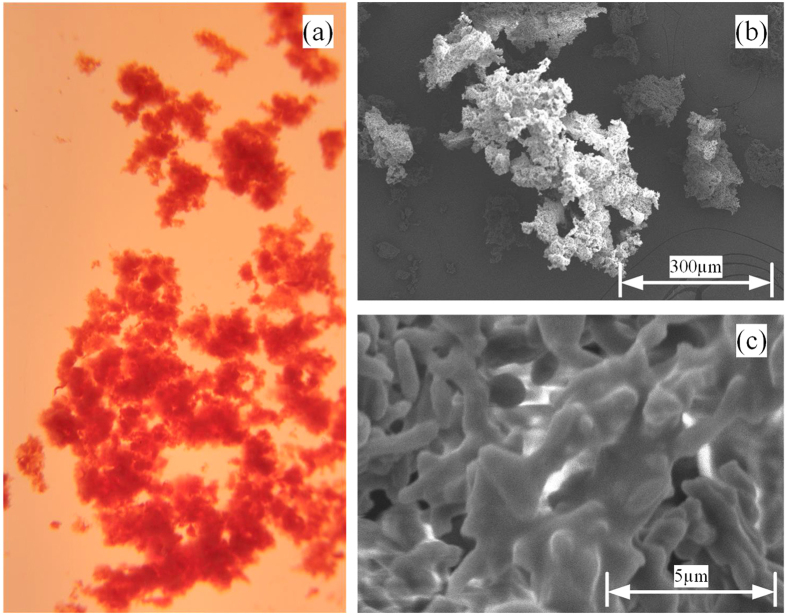
Bioaggregate of *R. faecalis* RLD-53 in photobioreactor at the optimum conditions. (**a**) Photo of bioaggregate; (**b**) and (**c**) SEM images of bioaggregate.

**Figure 7:**
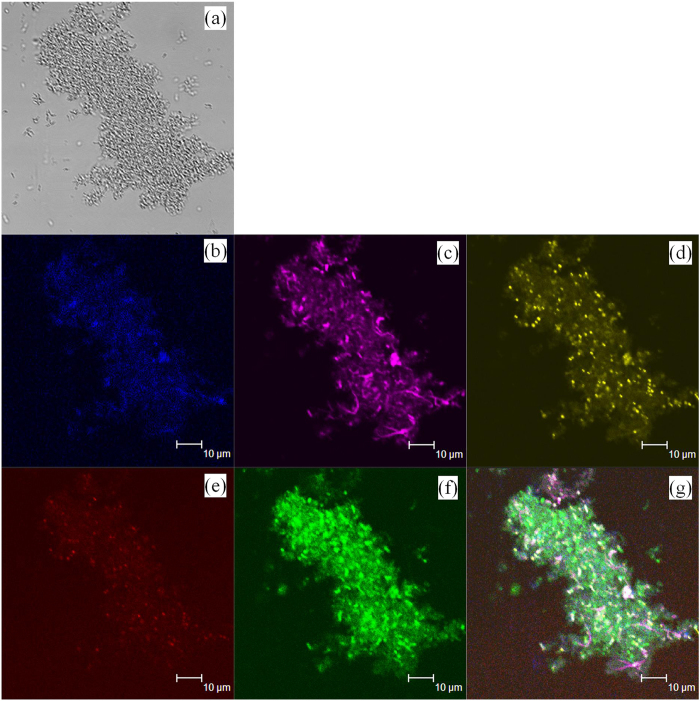
The CLSM images of bioaggregate of *R. faecalis* RLD-53 at the optimum conditions: (a), phase contrast photograph; (b), β-polysaccharides, light blue (calcofluor white), (c), proteins, violet (FITC), (d), lipids, yellow (Nile red); (e), α-polysaccharide, red (Con A rhodamine); (f), total cells, green (SYTO 63), (g), combined image of individual images in (b–f).

Bioflocculation is the process of microbial aggregation, which is critical to biomass/liquid separation^38^. In this study, the bioflocculation of PFB in the photobioreactor at the optimum conditions was shown in Fig. 6. It was evident that the PFB formed the bioaggregate with reddish-pink colour. SEM analysis also showed that bacteria cell formed a stable structure through covered and tightly linked together by EPS. Individual microbial flocs were composed of numerous bacterial cells, immobilized in matrices constituting polymers of proteins, polysaccharides, humic acids, and lipids. The distribution of these components in the bioflocculation was determined by staining the EPS and cells in bioaggregates (Fig. 7). According to the fluorescent intensity data presented in the Fig. 7, β-polysaccharides and proteins mainly accumulated at the outer layer of floc, whereas the lipids and α-polysaccharide evenly distributed in the floc. On the other hand, proteins were also within the core of the floc but to a lesser extent than the lips. With excellent settling abilities, photo-fermentative hydrogen producer gradually accumulated in the reactor and reached high concentration of 2.15 g/l.

Theoretically, PFB could convert 100% organic waste into hydrogen in the photobioreactor through harvesting energy from sunlight. However, photo-fermentation must consume a significant fraction of substrate for the production of biomass as biocatalysts, which decrease potential yield of the desired product^39^. Furthermore, PFB are stable suspension in the photobioreactor due to the poor flocculation, cannot be efficiently separated from supernatant and are washed out with effluent continuously. In the steady state of continuous operation, a considerable proportion of organic substrate have to be used for cell growth to mantain the biomass concentration at constant level. Hydrogen yields by growing PFB are relatively low as the vast majority of the electrons from organic feedstocks are used for biosynthesis^14^,^40^. As a result, the poor flocculation of PFB resulting in continuous washout of biomass leads to poor performance of the photobioreactor. In this study, stable floc of PFB induced by L-cysteine was applied in a photobioreactor operated in sequencing batch mode for enhancing hydrogen production. During settle stage of reactor operation, bacterial cells within the floc settled to the bottom of the reactor, rather than washed out with effluent. Consequently, a significant fraction of the reductant from organic substrate was diverted away from biomass synthesis into hydrogen production. Therefore, maximum hydrogen production rate 1044 ml/l/d and substrate conversion efficiency of 83.75% were obtained by PFB bioaggregate induced by L-cysteine in a sequencing batch photobioreactor.

In conclusion, this work presented a novel strategy to significantly enhance hydrogen recovery from organic wastes through bioflocculation of PFB induced by L-cysteine in a sequencing batch photobioreactor. During settling stage, bacterial cells within the floc were retained in the photobioreactor, and thereby most of the biomass avoided been washed out with effluent. This greatly improved the biomass retention capacity of the photobioreactor, which enabled the photobioreactor to be operated at a short HRT and high organic loading rate. As maximum biomass concentration achieved 2.10 g/l, the photobioreactor required more light energy at intensity of 200 W/m^2^. Under the optimum conditions, the maximum yield and rate of hydrogen production reached 3.35 mol H_2_/mol acetate and 1044 ml/l/d, respectively.

## Methods

### Bacterium and medium

A photo-fermentative bacterium, *Rhodopseudomonas faecalis* RLD-53 was used as the photo-hydrogen producer, which was isolated from fresh water pond sludge^41^. Acetate was used as the sole carbon source in the medium for hydrogen production. The culture medium of *R. faecalis* RLD-53 was prepared as described in previous report, and L-cysteine at 1 g/l was added to medium to induce bioflocculation formation^27^.

### Sequencing batch photobioreactor setup and operation

Continuous hydrogen production was conducted in 600 ml sequencing batch photobioreactor with 500 ml working volume (Fig. 1S, Supplementary information) at constant temperature of 35 ± 1 °C. The cylindrical photobioreactor was made of 2 mm glass with the inner diameter of 80 mm and height of 120 mm, respectively. The reactors were flushed 10 min using argon gas with high purity (99.99%) to maintain anaerobic conditions and autoclaved at 121 °C for 15 min. The liquid medium in the reactor was homogeneously mixed using a magnetic stirrer at 80 rpm to provide mixing during the feeding and reacting phases. Initially, *R. faecalis* RLD-53 in the mid-exponential growth phase was inoculated into reactors, which were operated in batch model for 4 days to accumulated biomass for continuous operation. After that, reactors were turned into sequencing batch operation including the following four sequential steps: Feed, React, Settle and Decant. The time used for each step was controlled by computer. The volume of Feed and Decant were 250 ml, half working volume of photobioreactor.

### Experiments of HRT affecting the performance of PFB bioaggregate

Hydrogen production was examined based on HRTs of 48, 96 and 144 h, corresponding to 1, 2, and 3 days per cycle, respectively. In this test, influent acetate concentration was kept consistent at 50 mmol/l. The light intensity on the outside surface of the reactors was maintained at 150 W/m^2^ by incandescent lamps (60 W). The operation times of the four sequential steps for the photobioreactor operation at various HRTs were shown in Table S1 (Supplementary information). All other operations were the same as described above. At each operation condition, the photobioreactor was operated over enough time to allow steady-state conditions.

### Experiments of influent concentration affecting the performance of PFB bioaggregate

To ensure a maximum hydrogen production and yield, three experimental runs were carried out at influent concentrations of 2.56, 3.84 and 5.12 g COD/l, while the system HRT was kept constant at 96 h. All other operations were the same as described above. At each operation condition, the photobioreactor was operated over enough time to allow steady-state conditions.

### Experiments of light intensity affecting the performance of PFB bioaggregate

In this test, hydrogen production performances were investigated at different of light intensity of 100, 200 and 300 W/m^2^, while the HRT and influent concentration were kept at 96 h and 3.84 g COD/l, respectively. All other operations were the same as described above. At each operation condition, the photobioreactor was operated over enough time to allow steady-state conditions.

### Structural analysis of PFB bioaggregate

Floc structure of the bioaggregate sample was evaluated by a scanning electron microscope^42^. The bioflocculation samples were fixed with 2.5% glutaraldehyde for 1.5 h in a 4 °C refrigerator. These samples were gently washed with phosphate buffer solution and then dehydrated by successive passages through 50%, 70%, 80%, 90%, and 100% ethanol. Each rinsing and dehydrating step took 10 min. The samples were refreeze dried (Hitachi E-2030, Japan) for 4 h, subsequently coated with gold powder by Sputter Coater (Hitachi E-1010, Japan) and finally attached on to the microscope supports with silver glue. Scanning electron microscope images were taken at 5 kV using an SEM (Hitachi S-3400N, Japan).

### Floc staining and confocal laser scanning microscopy imaging

Microbial EPS are biopolymers consisting of polysaccharides, proteins, nucleic acids and lipids, which have a significant influence on bioaggregate formation and maintain the structural integrity. The multiple color staining technique and confocal laser scanning microscope (CLSM) together used to identify the distribution of components in biological aggregates to understand the structure of floc. The staining process followed the procedures described by Adav *et al*.^43^. The collected flocs were maintained fully hydrated during staining. The dyes used in this study and their corresponding excitation and emission wavelengths were shown in Table S2 (Supplementary information). CLSM (Leica TCS-SP2 AOBS Confocal Spectral Microscope Imaging System, Germany) was used to visualize cell or EPS distributions in bioflocs. The confocal images obtained were processed and analysed using Image-Pro® plus 6.0 software (Media Cybernetics, Bethesda, MD, USA).

### Analytical methods

Light intensity was measured at the surface of reactor with solar power meter TENMARS TM-207 (Tenmars Electronics CO., LTD., Taiwan, China). Biogas was sampled from the head space of the photobioreactor by using gas-tight glass syringes and hydrogen content was determined by using a gas chromatograph (Agilent 4890D, Agilent Technologies, USA). Residual acetate in the effluent of the photobioreactor were determined using a second gas chromatograph (Agilent 7890 A, Agilent Technologies, USA) equipped with a flame ionization detector. The liquor samples were firstly centrifuged at 12,000 rpm for 5 min, and filtered through a 0.22 μm membrane before free acids were analyzed. The cell biomass was determined by filtering culture broth through a cellulose acetate membrane filter (0.45 μm pore size, 50 mm in diameter). After that, the filter was rinsed by deionized water to remove salts or non-cellular materials. Each loaded filter was dried at 105 °C until the weight became consistent. The dry weight of blank filter was subtracted from that of the loaded filter to obtain the cell biomass.

## Additional Information

**How to cite this article**: Xie, G.-J. *et al*. Bioaggregate of photo-fermentative bacteria for enhancing continuous hydrogen production in a sequencing batch photobioreactor. *Sci. Rep*. **5**, 16174; doi: 10.1038/srep16174 (2015).

## Supplementary Material

Supplementary Information
