# Supplementary material for: Bioaggregate of photo-fermentative bacteria for enhancing continuous hydrogen production in a sequencing batch photobioreactor
Source: Sci Rep. 2015 Nov 5;5:16174. doi: 10.1038/srep16174 (PMC4633638; doi:10.1038/srep16174)
Supplement: Supplementary Information [file srep16174-s1.doc]

**Supplementary information**

**Bioaggregate of photo-fermentative bacteria for enhancing continuous hydrogen production** **in a sequencing batch photobioreactor**

Guo-Jun Xie^1,2^, Bing-Feng Liu^1^,*, Rui-Qing Wang^1^, Jie Ding^1^, Hong-Yu Ren^1^, Xu Zhou^2^ and Nan-Qi Ren^1^,*

^1^State Key Laboratory of Urban Water Resource and Environment, Harbin Institute of Technology, Harbin 150090, China

^2^Advanced Water Management Centre, The University of Queensland, QLD 4072, Australia

*Corresponding author. E-mail: lbf@hit.edu.cn (B.F.L) or rnq@hit.edu.cn (N.Q.R); Tel/Fax: +86 451 86282008.

Table S1. Operating conditions of sequencing batch photobioreactors at different HRTs

| Parameters | HRT (h) | | |
| --- | --- | --- | --- |
|  | 48 | 96 | 144 |
| Cycle period (h) | 24 | 48 | 72 |
| Feed period (min) | 15 | 15 | 15 |
| React period (h) | 23 | 47 | 71 |
| Settle period (min) | 30 | 30 | 30 |
| Decant period (min) | 15 | 15 | 15 |
| Fill &decanting volume (ml) | 250 | 250 | 250 |

Table S2. The stains for bioaggregate staining and confocal laser scanning microscopy imaging

| Dye | Excitation (nm) | Emission (nm) | Targets in bioaggregate |
| --- | --- | --- | --- |
| Calcofluor white | 400 | 410–480 | β-1,4 and β-1,3 polysaccharides |
| FITC | 488 | 500–550 | Protein, amino-sugars |
| Nile red | 514 | 625–700 | Lipids, hydrophobic sites |
| Con A conjugates | 543 | 550–600 | α-Mannopyranosyl,α-glucopyranosyl sugars |
| Syto 63 | 633 | 650–700 | Total cells |


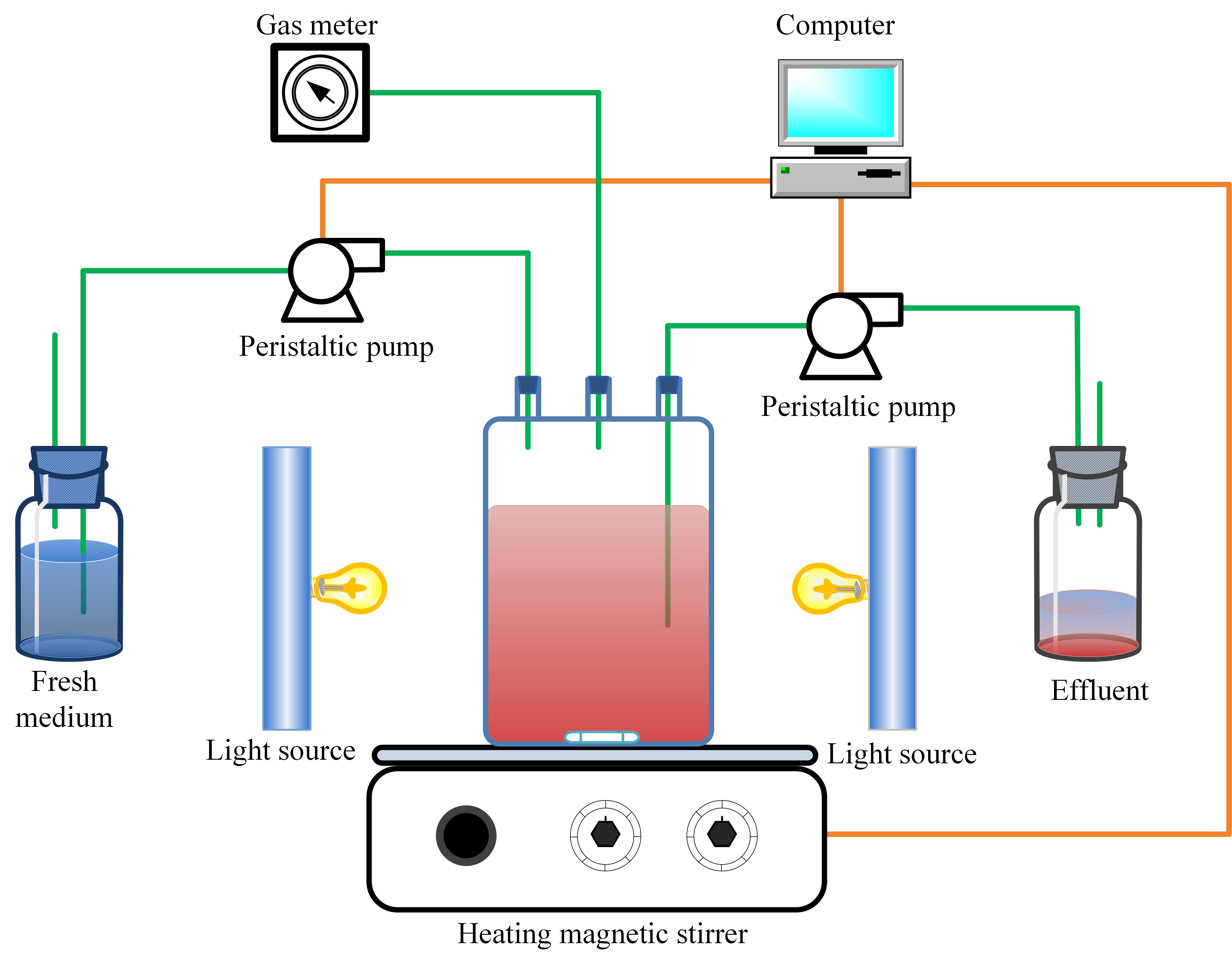


Figure S1. Schematic diagram of sequencing batch photobioreactor (Designed and drawn by first author, Dr Guo-Jun Xie).
